# Duodenoportal fistula caused by peptic ulcer after extended right hepatectomy for hilar cholangiocarcinoma

**DOI:** 10.1186/1477-7819-4-84

**Published:** 2006-11-24

**Authors:** Hiroyuki Kinoshita, Katsunari Takifuji, Yoshihiro Nakatani, Masaji Tani, Kazuhisa Uchiyama, Hiroki Yamaue

**Affiliations:** 1 Second Department of Surgery, Wakayama Medical University, School of Medicine, 811-1 Kimiidera, Wakayama 641-8510, Japan

## Abstract

**Background:**

A fistula between the duodenum and the main portal vein near a peptic ulcer is extremely rare, and only two cases of duodenal ulcers have been reported in the past.

**Case presentation:**

We report a 68-year-old man with a diagnosis of anemia who had a history of extended right hepatectomy for hilar cholangiocarcinoma 20 months previously. The first endoscopic examination revealed a giant peptic ulcer with active bleeding at the posterior wall of the duodenal bulbs, and hemostasis was performed. Endoscopic treatment and transarterial embolization were performed repeatedly because of uncontrollable bleeding from the duodenal ulcer. Nevertheless, he died of sudden massive hematemesis on the 20^th ^hospital day. At autopsy, communication with the main portal vein and duodenal ulcer was observed.

**Conclusion:**

It should be borne in mind that the main portal vein is exposed at the front of the hepatoduodenal ligament in cases with previous extrahepatic bile duct resection.

## Background

The numbers of reported cases of fistula between the portal venous system and adjacent organs has been gradually increasing recently. For instance, fistula has been reported between the portal venous system and the pancreas [1,2], biliary system [3,4], small intestine [5-7], and colon [8,9]. However, fistula between the main portal vein and duodenum is extremely rare, with only three reported cases [5-7]. Herein, we report an extremely rare case of a fistula between the main portal vein and duodenum after resection of extrahepatic bile duct due to hilar cholangiocarcinoma, and we discuss the strategy of duodenal peptic ulcer with massive bleeding.

## Case presentation

**Figure 1:**
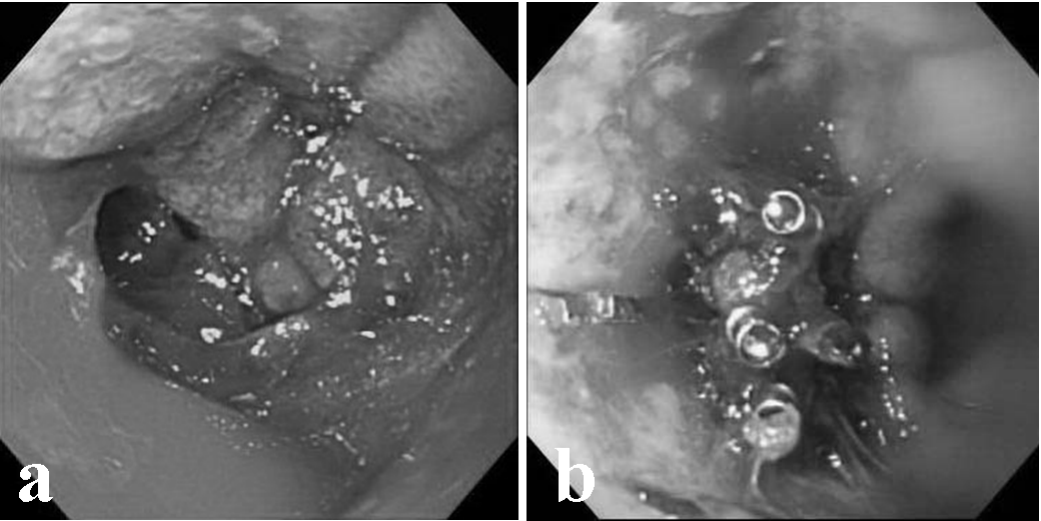
a) Duodenoscopy showed a giant peptic ulcer with active bleeding at the posterior wall of the duodenal bulbs. 1b) Endoscopic therapy by clips was performed for recurrent bleeding again six days after the first TAE.

**Figure 2:**
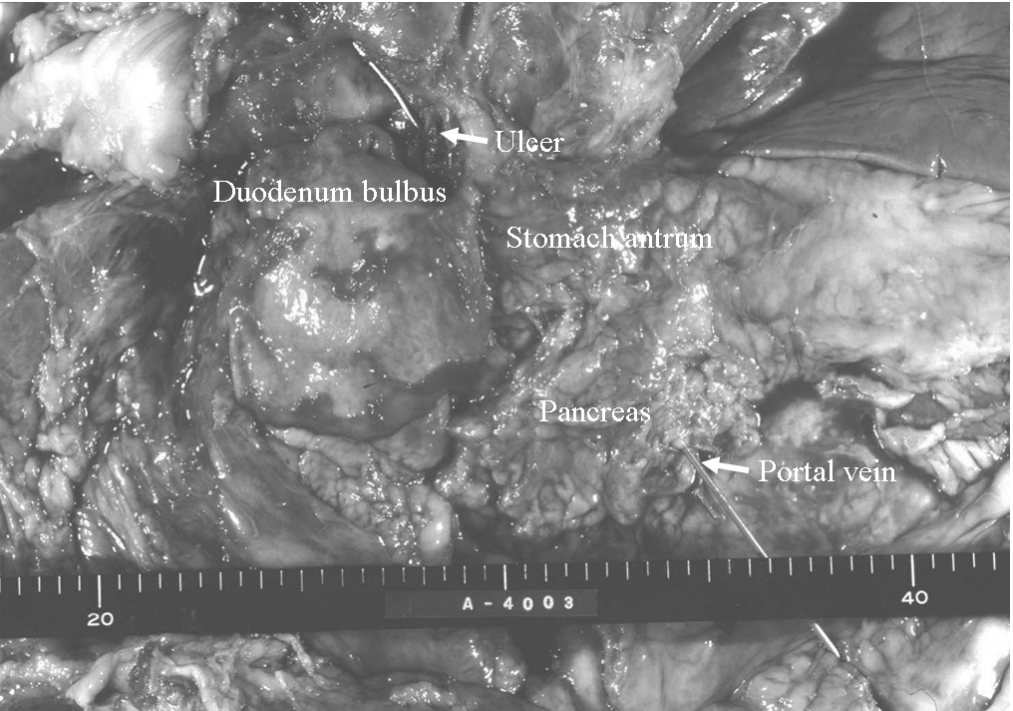
The communication with the main portal vein and duodenal ulcer was manifested by insertion of a stick, at autopsy.

A 68-year-old man with a diagnosis of anemia was admitted to Wakayama Medical University Hospital. He had a history of extended right hepatectomy for hilar cholangiocarcinoma (stage 2) 20 months earlier. On arrival, his blood pressure and pulse rate were 99/54 mm Hg and 101/min, respectively. Initial laboratory studies showed his hematocrit and hemoglobin were 25.8% and 8.2 g/dL. Nasogastric tube lavage revealed a material that looked like coffee grounds. Emergent gastrointestinal endoscopic examination was performed immediately and disclosed a giant peptic ulcer with active bleeding at the posterior wall of the duodenal bulbs (Figure 1a). Angiography was performed for the bleeding duodenal ulcer, which was not controlled by endoscopic hemostasis. Extravasation of contrast medium was not noted at the artery around the duodenum; however, transarterial embolization (TAE) was accomplished for the duodenal branch of the gastroduodenal artery. Massive hematemesis and anal bleeding occurred on the sixth day after the first TAE. The bleeding point of the ulcer was endoscopically treated with a clip (Figure 1b). Nevertheless, because bleeding from the duodenal ulcer occurred repeatedly, endoscopic treatment and TAE was performed, and endoscopic hemostasis was performed on the 10^th^, 14^th^, and 17^th ^day, and TAE was performed on the 10^th^, 14^th^, 19^th ^day. Finally, we embolized the common hepatic artery, the bilateral subphrenic artery, and the jejunal branch for hepaticojejunostomy. However, the patient died of sudden massive hematemesis on the 20^th ^hospital day. At autopsy, a peptic ulcer measuring 1.5 cm was present in the bulbus of the duodenum. The communication with the main portal vein and duodenal ulcer was manifested by insertion of a stick (Figure 2).

## Discussion

To our knowledge, only two cases have been reported that involved a penetrating ulcer of the duodenal bulbus extending into the main portal vein forming a fistula [6,7]. Povoski *et al*. [7] reported a case with a fistula involving the portal vein and the duodenum at the site of a duodenal ulcer in a patient who had undergone previous extrahepatic bile duct resection and brachytherapy, which is similar to our case. They were successful in directly closing the fistula in the anterior wall of the portal vein after they took down the antecolic hepaticojejunostomies and divided the duodenal bulbus. Soares *et al*. [6] presented a case with a fistula between the duodenum and the portal vein caused by peptic ulcer with no history of previous surgery. They explained there was a small window superior to the pancreas and between the gastroduodenal artery and common bile duct where the portal vein and duodenum are separated by adipose tissue without pancreatic interposition (Additional file 1).

Bleeding is a serious complication of duodenal ulcers. Endoscopic hemostasis is the established first-line method for bleeding duodenal ulcers. The primary technical success rate is reportedly 90% in most studies [10]. However, recurrent bleeding has an incidence of about 15% [11]. If hemostasis is not achieved by endoscopic treatment, TAE or surgery may be required. However, controversy exists as to the safety and efficacy of these methods. Some authors have asserted that TAE is an effective method to stop massive bleeding from gastroduodenal ulcers in a high percentage of patients [12,13]. On the other hand, there are some reports that early elective surgery should be recommended in high-risk patients with bleeding duodenal bulb ulcer after failure of primary endoscopic treatment [14]. The rates for emergency surgery are reportedly about 10% in general [15].

In the present case, because bleeding from the duodenal ulcer occurred repeatedly, endoscopic treatment and TAE were performed on all occasions without surgery, in expectation of the presence of numerous adhesions in the surrounding area of the mobilized duodenum.

There are few reports employing metallic stents in patients with malignant stenosis of the portal vein [16]. In our case, if it had been revealed that the duodenal ulcer penetrated into the portal vein by extravascular extravasation of contrast medium in percutaneous transhepatic portography, the placement of the covered metallic stent may have been an effective modality with apprehension of patancy, before the repeated endoscopic and interventional radiologic therapy.

## Conclusion

We should keep in mind that the main portal vein is exposed at the front of the hepatoduodenal ligament in cases with previous extrahepatic bile duct resection.

## Competing interests

The author(s) declare that they have no competing interests.

## Authors' contributions

**HK **designed the study and participated in writing process. **KT **was the main endoscopist managing the case. **YN **designed the study and collected the clinical data. **MT **helped to draft the manuscript.

**KU **participated in the editing process. **HY **revised the manuscript.

All authors read and approved the final manuscript.

## Supplementary Material

Additional file 1 Published cases of duodenoportal fistula Click here for file

## References

[B1] Willis SM, Brewer TG (1989). Pancreatic duct-portal vein fistula. Gastroenterology.

[B2] Lum C, Cho KC, Scholl DG, Sundaram NK (1998). Portal vein opacification during ERCP in patients with pancreatitis. Abdom Imaging.

[B3] Antebi E, Adar R, Zweig A, Barzilay J, Mozes M (1973). Bilemia: an unusual complication of bile duct stones. Ann Surg.

[B4] Lugagne P-M, Lacaine F, Bonnel D, Ligory C, Huguier M (1988). Bilioportal fistula as a complication of choledochoduodenostomy. Surgery.

[B5] Dingeldein GP, Proctor HJ, Jaques PF (1977). Traumatic aorto-caval-portal-duodenal fistula. Case report. J Trauma.

[B6] Soares MA, Wanless IR, Ambus U, Cameron R (1996). Fistula between duodenum and portal vein caused by peptic ulcer disease and complicated by hemorrhage and portal vein thrombosis. Am J Gastroenterol.

[B7] Povoski SP, Shamma JM (2003). Fistula involving portal vein and duodenum at the site of a duodenal ulcer in a patient after previous extrahepatic bile duct resection and brachytherapy. Dig Surg.

[B8] Rothman BJ, Cloogman H, Wong D (1981). Colovenous fistula complicating diverticulitis. Am J Gastroenterol.

[B9] Sonnenshein MA, Cone LA, Alexander RM (1986). Diverticulitis with colovenous fistula and portal venous gas. J Clin Gastroenterol.

[B10] Buffoli F, Graffeo M, Nicosia F, Gentile C, Cesari P, Rolfi F, Paterlini A (2001). Peptic ulcer bleeding: Comparison of two hemostatic procedures. Am J Gastroenterol.

[B11] Lin HJ, Tseng GY, Lo WC, Lee FY, Perng CL, Chang FY, Lee SD (1998). Predictive factors for rebleeding in patients with peptic ulcer bleeding after multipolar electrocoagulation: a retrospective analysis. J Clin Gastroenterol.

[B12] Kramer SC, Gorish J, Rilinger N, Siech M, Aschoff AJ, Vogel J, Brambs HJ (2000). Embolization for gastrointestinal hemorrhages. Eur Radiol.

[B13] Ljungdahl M, Eriksson LG, Nyman R, Gustavsson S (2002). Arterial embolization in management of massive bleeding from gastric and duodenal ulcer. Eur J Surg.

[B14] Monig SP, Lubke T, Baldus SE, Schafer H, Holscher AH (2002). Early elective surgery for bleeding ulcer in the posterior duodenal bulb. Own results and review of the literature. Hepatogastroenterology.

[B15] Guglielmi A, Russenente A, Sandri M, Kind R, Lombardo F, Rodella L, Catalano F, de Manzoni G, Cordiano C (2002). Risk assessment and prediction of rebleeding in bleeding gastroduodenal ulcer. Endoscopy.

[B16] Tanaka J, Andoh H, Yoshioka M, Furuya T, Asanuma Y, Koyama K (2000). Palliative treatment with metallic stents for unresectable gallbladder carcinoma involving the portal vein and bile duct. J Hepatobiliary Pancreat Surg.

