# Supplementary figures and images for: Duodenoportal fistula caused by peptic ulcer after extended right hepatectomy for hilar cholangiocarcinoma
Source: World J Surg Oncol. 2006 Nov 24;4:84. doi: 10.1186/1477-7819-4-84 (PMC1676009; doi:10.1186/1477-7819-4-84)

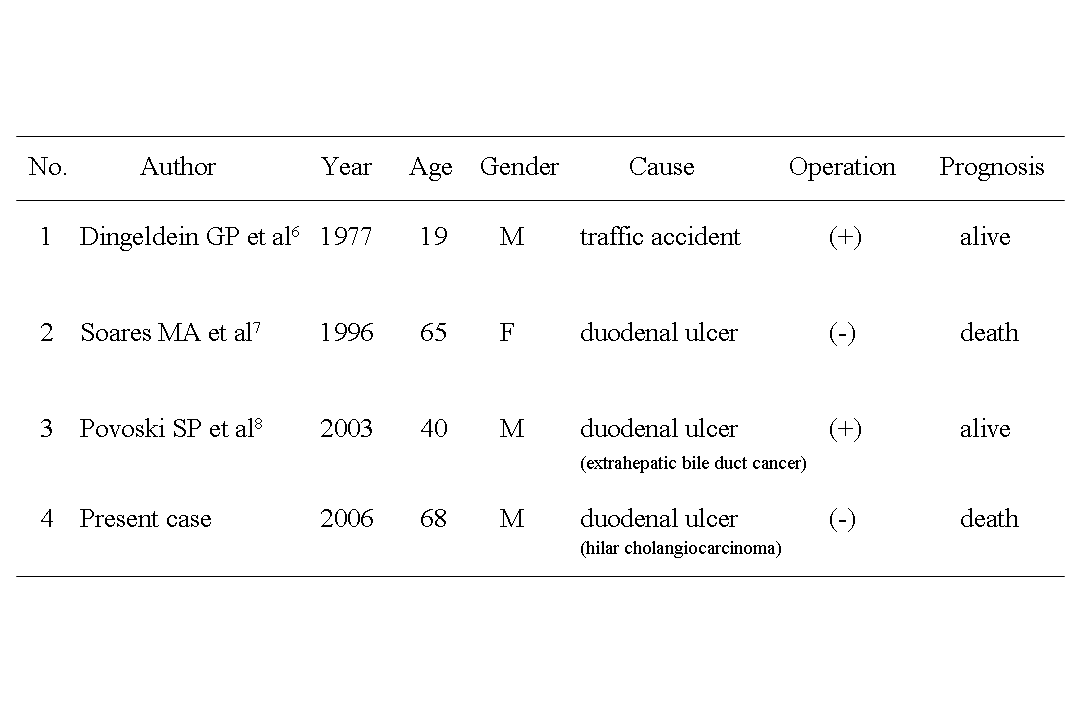

Supplement: Additional file 1 — Published cases of duodenoportal fistula [file 1477-7819-4-84-S1.tiff]
